# Supplementary material for: The Diagnostic Value of microRNA Expression Analysis in Detecting Intraductal Papillomas in Patients with Pathological Nipple Discharge
Source: Int J Mol Sci. 2024 Feb 2;25(3):1812. doi: 10.3390/ijms25031812 (PMC10855314; doi:10.3390/ijms25031812)
Supplement: Supplementary file 1 [file ijms-25-01812-s001.zip › ijms-2748630-supplementary.pdf]

Positive if Greater Than or Equal To[a]	Sensitivity	1 - Specificity
,0000000	1,000	1,000
,0133635	1,000	,857
,0330240	1,000	,714
,1018290	1,000	,571
,1794431	1,000	,429
,3284698	1,000	,286
,5064091	,938	,286
,6159065	,875	,286
,6968089	,813	,286
,7436575	,750	,286
,7952032	,750	,143
,8234184	,688	,143
,8305200	,688	,000
,9045949	,625	,000
,9792028	,563	,000
,9849318	,500	,000
,9859012	,438	,000
,9871177	,375	,000
,9908372	,313	,000
,9967484	,250	,000
,9996004	,188	,000
,9999713	,125	,000
,9999824	,063	,000
1,0000000	,000	,000

a. The smallest cutoff value is the minimum observed test value minus 1, and the largest cutoff value is the maximum observed test value plus 1. All the other cutoff values are the averages of two consecutive ordered observed test values.

Supplementary Table S1: The coordinates of the ROC curve using the predicted probabilities

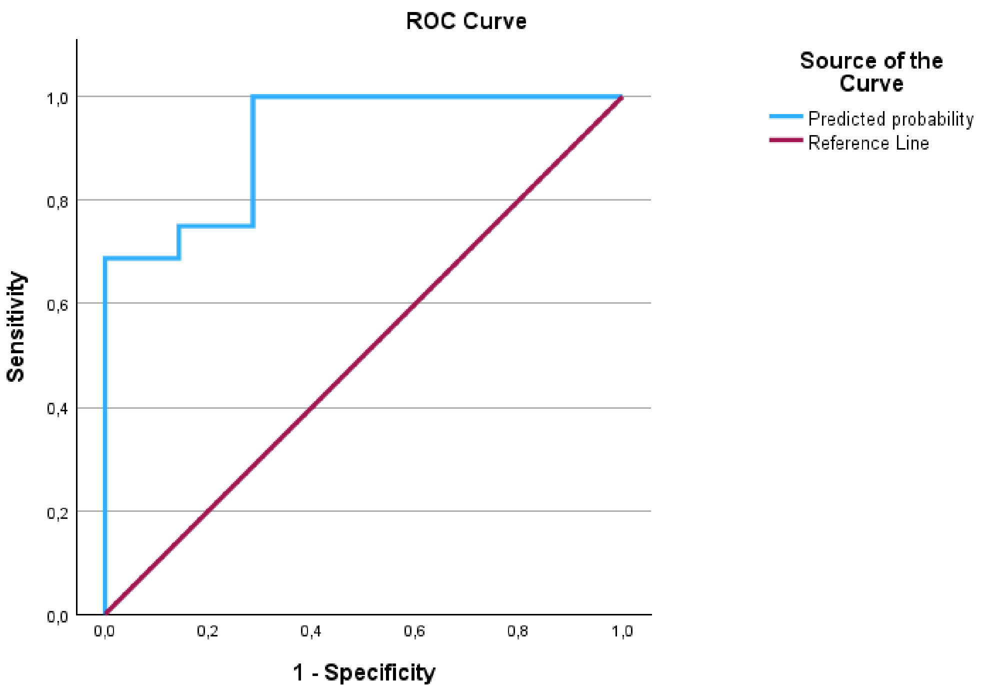

Supplementary Figure S1: Receiver operating characteristic (ROC) curve of microRNA miR-145-5p is shown demonstrating its accuracy rate of 0.920 (CI 0.801 – 1.000, p=0.002) in distinguishing benign from intraductal papillomas in pathological nipple discharge samples.
